# Locally advanced triple negative breast cancer arising from fibroadenoma with complete response to neoadjuvant chemotherapy: A case report

**DOI:** 10.1016/j.ijscr.2020.02.059

**Published:** 2020-02-29

**Authors:** Sho Shiino, Masayuki Yoshida, Momoko Tokura, Chikashi Watase, Takeshi Murata, Kenjiro Jimbo, Shin Takayama, Akihiko Suto, Kaishi Satomi, Akiko Miyagi Maeshima, Mari Kikuchi, Nachiko Uchiyama, Takayuki Kinoshita

**Affiliations:** a Department of Breast Surgery, National Cancer Center Hospital, Tokyo 104-0045, Japan; b Department of Diagnostic Pathology, National Cancer Center Hospital, Tokyo 104-0045, Japan; c Diagnostic Imaging Department, Cancer Institute Hospital, Tokyo 135-8550, Japan; d Department of Diagnostic Radiology, National Cancer Center Hospital, Tokyo 104-0045, Japan; e National Hospital Organization Tokyo Medical Center, Tokyo 152-8902, Japan

**Keywords:** FA, fibroadenoma, IDC, invasive ductal carcinoma, HR, hormone receptor, TN, triple negative, LN, lymph node, pCR, pathological complete response, T, tesla, MRI, magnetic resonance imaging, PET-CT, positron emission tomography-computed tomography, FDG, ^18^F-fluorodeoxyglucose, SUV, standardized uptake value, CEA, carcinoembryonic antigen, CA15-3, cancer antigen 15-3, CNB, core needle biopsy, ER, estrogen receptor, PgR, progesterone receptor, HER2, human epidermal growth factor receptor-2, NAC, neoadjuvant chemotherapy, PR, partial response, Case report, Fibroadenoma, Invasive ductal carcinoma, Triple negative, Pathological complete response, Neoadjuvant chemotherapy

## Abstract

**Introduction:**

Breast cancer arising from benign fibroadenoma (FA) is rare. The histological type of the former was either carcinoma *in situ* or early-stage invasive breast carcinoma with hormone receptor positive/HER2 (human epidermal growth factor receptor-2)-negative phenotype. Meanwhile, advanced breast cancer of triple negative (TN) phenotype such as our case is extremely uncommon and clinically challenging.

**Presentation of case:**

We experienced a case of a 53-year-old woman that had invasive ductal carcinoma of TN phenotype in FA with multiple lymph node metastases. After receiving neoadjuvant chemotherapy (NAC), she underwent breast mastectomy and axillary dissection. The pathological examination on postoperative specimens revealed the dense fibrous stroma in the FA without any residual viable tumor cells and was considered as pathological complete response (pCR).

**Discussion:**

This is the first report presenting a case of NAC treatment for invasive ductal carcinoma (IDC) in FA. Furthermore, the patient achieved pCR even if IDC was located within FA. Diagnosing breast cancer in FA may be challenging as the carcinoma component may be hidden by the FA component. If imaging of FA became larger or abnormal changes during follow-up examinations, needle biopsy should be recommended for assessment of the lesion positively.

**Conclusion:**

This is the first report presenting a case of advanced breast cancer in FA of TN phenotype with multiple lymph node metastases who achieved pCR even if IDC was located within FA.

## Highlights

• Breast cancer arising in association with a fibroadenoma (FA) is rare and usually early-stage.
• We report a rare case of an advanced triple negative breast cancer, with lymph node metastases, within a long-standing FA.
• Pathological complete response was achieved after neoadjuvant chemotherapy, as proven on mastectomy with axillary clearance.

## 1 Introduction

Fibroadenoma (FA) is a common benign tumor of the breast. In most cases, FA shows a benign clinical course, although breast carcinoma can occasionally develop in FA. Most of the histological type is *in situ* malignancy, but invasive breast carcinoma can develop in FA [1,2].

Knowledge of the specific tumor subtype is important to determine the appropriate preoperative and postoperative drug therapy. Previous studies have reported a better survival prognosis for breast cancer developing within an FA than the common types of breast cancer [3,4], because many of the former are hormone receptor (HR) positive and their histological type is mainly carcinoma *in situ* and early-stage [1]. A triple negative (TN) phenotype is extremely uncommon [4]. Here, we report a case of a locally advanced TN breast cancer developing within an FA with multiple local lymph node (LN) metastases, where a pathological complete response (pCR) was achieved. This case has been reported in line with the SCARE criteria [5].

## 2 Presentation of case

A 53-year-old woman had noticed a non-painful lump in her right breast since she was 40 years old. She underwent mammography and ultrasonography for examination of the tumor, which was clinically diagnosed as benign. After that, she underwent follow-up examinations for almost 10 years during which time the tumor showed no apparent changes. The patient did not undergo a core biopsy for the original tumor during follow-up examination because the tumor was clinically diagnosed as benign. After stopping the follow-up examinations for 2 years, she noticed an enlarging mass in the same area and decided to visit our hospital. Physical examination revealed a palpable and hard mass, 2.5 cm in diameter, in the right inner and lower quadrant area of her breast.

**Fig. 1:**
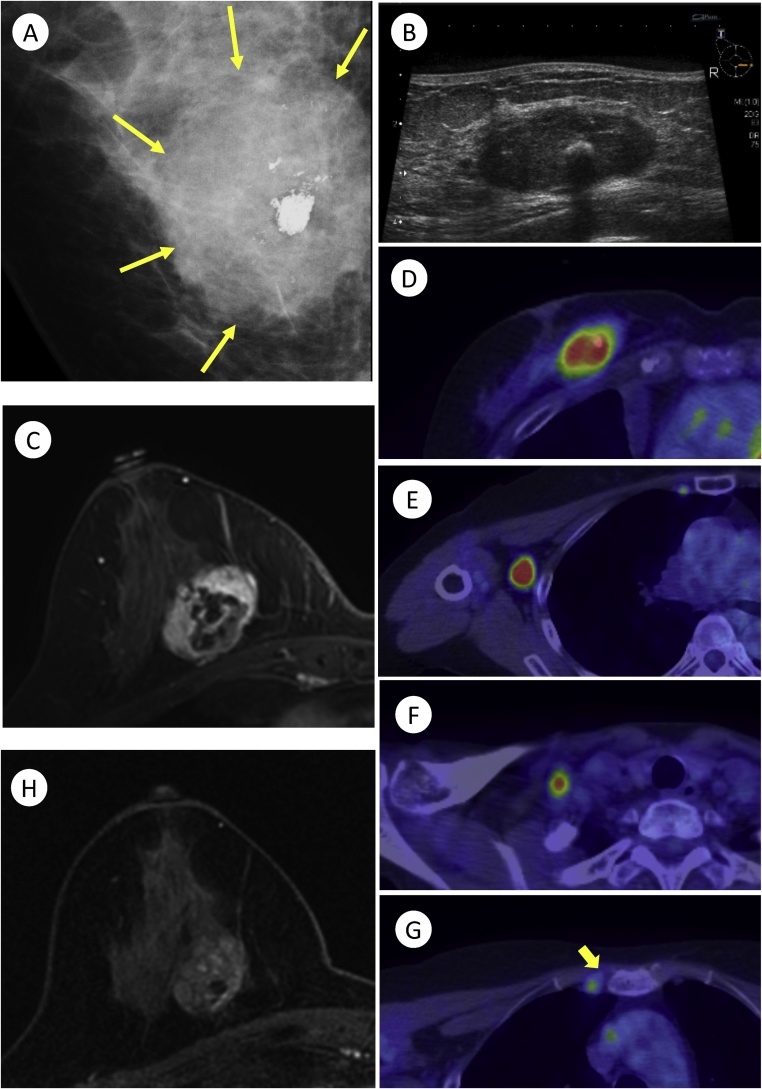
Pre-NAC imaging examinations of the right breast tumor (Fig. 1A–G). A: Mammography showed a solitary mass with ill-defined margins, a coarse calcification, and pleomorphic calcifications (yellow arrow). B: Ultrasonography showed a solitary mass with smooth or irregular margins. C: Dynamic contrast-enhanced T1-weighted 3.0 T magnetic resonance imaging (MRI) with fat suppression (90 s) showed a 3.6-cm oval mass with partially ill-defined and markedly enhanced margins. High FDG uptake for right breast tumor (D), regional lymph node (E), supraclavicular lymph node (F), and internal mammary lymph node (G; yellow arrow) in positron emission tomography-computed tomography (PET-CT) imaging. Post-NAC imaging examinations of the right breast tumor (Fig. 1H). H: 3.0 T MRI (90 s) after receiving neoadjuvant chemotherapy showed the remaining 2.7-cm tumor with a reduction in size and decreased enhancement.

Mammography showed a solitary mass with ill-defined margins containing a coarse calcification with pleomorphic calcifications around it in the same area (Fig. 1A). Ultrasonography examination also showed a heterogeneous and solitary mass with smooth or irregular boarders (Fig. 1B). Additionally, enlarged LNs in her right axillary area and supraclavicular area were noted. Contrast-enhanced 3.0 Tesla (T) magnetic resonance imaging (MRI) also showed a 3.6-cm oval mass with partially ill-defined margins and a heterogeneous internal enhancement in her right breast (Fig. 1C). A dynamic contrast-enhanced curve showed a fast enhancement (90 s) with markedly enhanced margins, followed by either a washout or a persistent pattern at the different areas. Positron emission tomography-computed tomography (PET-CT) imaging demonstrated ^18^F-fluorodeoxyglucose (FDG) uptake (maximum standardized uptake value [SUVmax]: 7.14) by the same lesion as well as in the axillary, supraclavicular, and internal mammary LNs (Fig. 1D–G). No distant metastases were found on PET-CT. Laboratory examination revealed normal carcinoembryonic antigen (CEA) and cancer antigen 15-3 (CA15-3) levels. Neither the patient nor her family had a history of malignant tumors, which includes breast cancer.

**Fig. 2:**
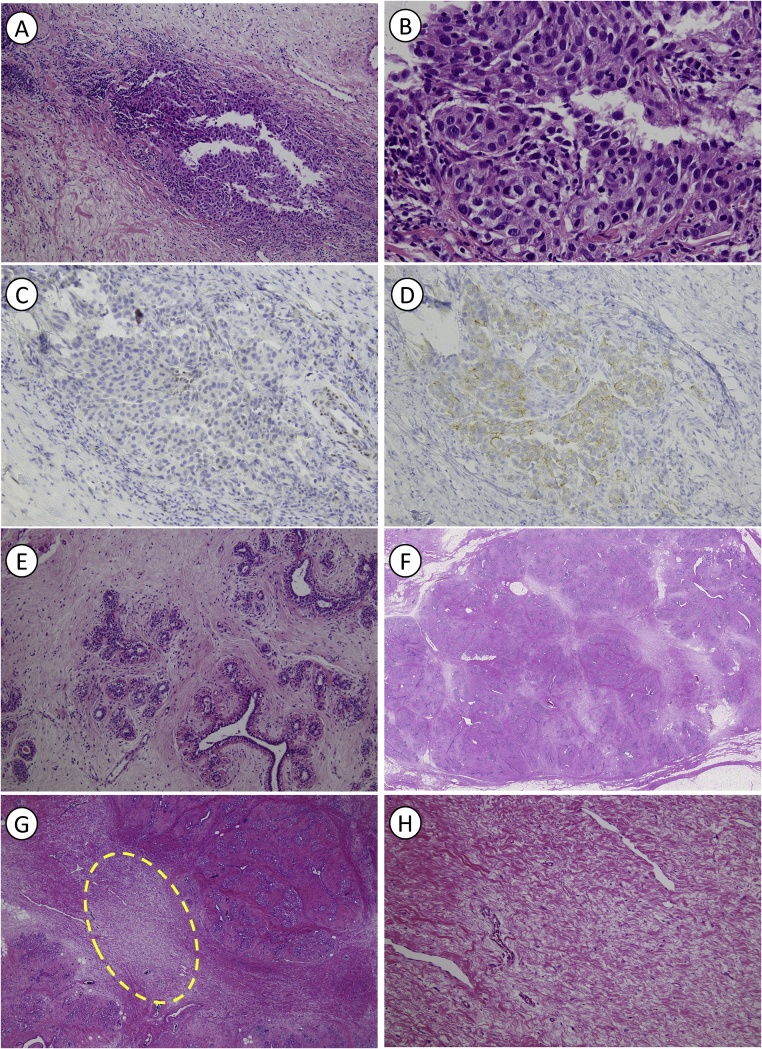
Histological and immunohistochemical findings of the tumor. A, B: Invasive ductal carcinoma detected *via* core needle biopsy (A: hematoxylin and eosin staining, ×100; B: hematoxylin and eosin staining, ×400). Immunohistochemical analysis of the tumor showed absence of estrogen receptor (C) and human epidermal growth factor receptor-2 (D). E: Well-developed terminal duct lobular unit-like structures in the core needle biopsy specimens (hematoxylin and eosin staining, ×100). F: A well-demarcated, solid mass which suggests organoid type FA (hematoxylin and eosin staining, scanning magnification). G: Fibroadenoma with fibrous scar (yellow loop), which was considered as post therapeutic effect (hematoxylin and eosin staining, ×20). H: Fibrous scar in the tumor (hematoxylin and eosin staining, ×200).

The tumor was pathologically diagnosed as invasive ductal carcinoma (IDC) using core needle biopsy (CNB), which was histological grade 3 (Fig. 2A, B). Infiltrating tumor cells showed high-grade nuclear atypia and a solid growth pattern. No expression of estrogen receptor (ER), progesterone receptor (PgR), or human epidermal growth factor receptor-2 (HER2) was observed, indicating a triple negative phenotype (Fig. 2C: Expression of ER, Fig. 2D: Expression of HER2). The antibodies used were mouse monoclonal anti-ER antibody (clone 1D5; Dako, Glostrup, Denmark), mouse monoclonal anti-PR antibody (clone PgR636, Dako), and rabbit polyclonal anti-HER2 antibody (HercepTest, Dako). In addition, the Ki-67 (MIB-1, Dako monoclonal mouse antibody, Agilent Technologies, Tokyo, Japan) labeling index was 75.8%. The parenchyma of the CNB specimen was surrounded by a dense population of well-developed terminal duct lobular unit-like structures (Fig. 2E). As only a few of these structures were present, this tumor could not be diagnosed as FA based only on the CNB specimen. Metastatic adenocarcinoma was detected by fine needle aspiration of axillary enlarging LNs.

The patient received neoadjuvant chemotherapy (NAC) with adriamycin and cyclophosphamide, followed by weekly administration of paclitaxel. A 3.0 T contrast-enhanced MRI after NAC treatment showed the remaining 2.7-cm mass with a reduction in size and decreased enhancement (Fig. 1H). The FDG uptake of the primary lesion decreased (SUVmax: 1.98) but was still higher than normal. Therefore, we judged the therapeutic effect as partial response (PR). In contrast, FDG uptake was negative for the regional lymph node. After NAC, we performed breast mastectomy and axillary dissection for this patient.

The pathological examination on operative specimens revealed a well-demarcated, solid mass macroscopically (Fig. 2F). Histologically, the tumor consisted mainly of the stroma proliferated around tubular ducts. No atypia and mitosis were noted on either stromal or ductal cells, suggesting organoid type FA. Additionally, we found the dense fibrous stroma in the FA without any residual viable invasive tumor cells (Fig. 2G). This histological finding was similar with post-therapeutic change and was considered as pCR. The scar was primarily located in the central area of the FA, and no scar was found in the adjacent breast parenchyma (Fig. 2H). Fibrous scar tissue was also detected in one of the axillary LNs, but no viable tumor cells were found.

After surgery, the patient underwent radiation therapy (50 Gy in 25 fractions) at her chest wall and regional LN including the supraclavicular and internal mammary areas. She had no lesion recurrence for approximately 2.5 years.

## 3 Discussion

This is the first report presenting a case of NAC treatment for IDC in FA of TN phenotype in a patient with multiple LN metastases who achieved pCR even if IDC was located within FA. According to previous reports, the histological type of breast carcinoma in FA was mainly carcinoma *in situ*, followed by invasive carcinoma [1,2,6]. IDC in FA is very rare with an incidence of 0.02%–0.125% [7,8]. The rate of HR and HER2 expression of IDC in FA was about 68.8% in ER, 62.5% in PgR, and 10.0% in HER2 [4], although the tumor subtype was not described in detail. Our case had TN phenotype, and this IDC subtype in FA would be considered as extremely rare. Moreover, IDC in FA with axillary LN metastases is unusual [4]. A previous study has already reported two cases of IDC in FA with multiple LN metastases [9]. However, both cases were HR-positive phenotype, which was the most common phenotype. Advanced breast cancer of TN phenotype with multiple LN metastases such as our case have not been reported previously.

Some studies suggested that women with FA have 1.7–2.17 times higher risk of developing breast cancer than women without FA [10,11]. Meanwhile, Ciatto et al. reported that the risk assessment of breast cancer, which was subsequent to the diagnosis of FA, was due to an existing bias [12]. Although these findings indicate the increasing risk of developing IDC in breast parenchyma outside of FA, the risk for developing IDC in FA remains unclear.

Some studies suggested that the imaging features of carcinoma in FA were as follows: large in size, irregular in shape, have indistinct margins, and have abnormal calcifications (linear, pleomorphic, or microcalcifications) [13,14]. The features (partially ill-defined margins, abnormal calcifications, and larger in size) reported in our case study were compatible with those of previous studies. However, enhanced MRI showed heterogeneous internal enhancement pattern and fast washout area in the tumor. As these features were similar with the imaging features of breast cancer of TN phenotype [15], we could not accurately evaluate if the tumor consisted of both IDC and FA.

In the event of IDC development in FA, diagnosing it as IDC in FA may be challenging as the carcinoma component may be hidden by the FA component and, therefore, may not be detected by imaging examination. As the tumor of our case became larger and mammography showed multiple abnormal calcifications, we recommended CNB in order to detect breast cancer histologically. If imaging of FA showed enlargement or abnormal changes during follow-up examinations, a CNB should be performed positively. Meanwhile, Wu et al. reported that mean age at diagnosis of breast cancer in FA is typically 46.9 years, which is higher than the age at diagnosis of benign FA (20–30 years) [4]. When patients aged about 40 years and older have clinically benign FA on imaging examinations, clinicians need to discuss with patients at least once whether a CNB should be performed.

Healed sites of previous IDC can be determined by architectural distortion characterized by fibrosis, stromal edema, increased vascularity composed largely of thin-walled vessels, and a chronic inflammatory cell infiltrate [16]. Stroma of FA is sometimes fibrous, especially in patients who suffered FA for a long time (so-called ancient FA). In the present case, we could assess the therapeutic effect of NAC treatment, as there were uniform fibrous areas with low cellularity that did not resemble other stromal areas of the FA. Histological careful observation could distinguish a fibrous tumor bed from the original stroma of FA.

## 4 Conclusion

This is the first report presenting a rare case of advanced breast cancer in FA of TN phenotype with multiple LN metastases, in which pCR was achieved after NAC.

## Sources of funding

The present study did not receive any specific grant from funding agencies in the public, commercial, or non-for-profit sectors.

## Ethical approval

The present study was approved by the internal review board of the National Cancer Center, Tokyo, Japan (no. 2012-278).

## Consent

Written informed consent was obtained from the patient for the publication of the case report. The patient share her perspective on the treatments she received.

## Author contribution

MY: corresponding author/ collected the data and interpreted the data.

SS: first author/ the surgeon who prepared the patient, planned the surgery, run the operation, collected the data and interpreted the data, and wrote the paper.

MT, CW, TM, KJ, ST, AS, KS, AMM, MK, NU, and TK: co-author/ interpreted the data.

## Registration of research studies

Our manuscript is a case report, not a research.

## Guarantor

Masayuki Yoshida.

Sho Shiino.

## Provenance and peer review

Not commissioned, externally peer-reviewed.

## Declaration of Competing Interest

We don’t have any conflicts of interest.
